# Safety out of control: dopamine and defence

**DOI:** 10.1186/s12993-016-0099-7

**Published:** 2016-05-23

**Authors:** Kevin Lloyd, Peter Dayan

**Affiliations:** Gatsby Computational Neuroscience Unit, 25 Howland Street, London, UK

**Keywords:** Dopamine, Defence, D_2_ receptor, Striatum

## Abstract

We enjoy a sophisticated understanding of how animals learn to predict appetitive outcomes and direct their behaviour accordingly. This encompasses well-defined learning algorithms and details of how these might be implemented in the brain. Dopamine has played an important part in this unfolding story, appearing to embody a learning signal for predicting rewards and stamping in useful actions, while also being a modulator of behavioural vigour. By contrast, although choosing correct actions and executing them vigorously in the face of adversity is at least as important, our understanding of learning and behaviour in aversive settings is less well developed. We examine aversive processing through the medium of the role of dopamine and targets such as D_2_ receptors in the striatum. We consider critical factors such as the degree of control that an animal believes it exerts over key aspects of its environment, the distinction between ‘better’ and ‘good’ actual or predicted future states, and the potential requirement for a particular form of opponent to dopamine to ensure proper calibration of state values.

## Background

Our comprehension of appetitive Pavlovian and instrumental conditioning at multiple levels of theory and experiment has progressed dramatically over the last few years. We now enjoy a richly detailed picture, encompassing computational questions about the sorts of prediction and optimization that animals perform, and priors over these; algorithmic issues about the nature of different sorts of learning that get recruited and exploited in various circumstances; and implementational details about the involvement of many structures, including substantial pre-frontal cortical areas, the amygdala, the striatum, and also their respective dopaminergic neuromodulation [1–8]. Along with this evolving understanding of discrete choice, there is evidence that the vigour of engagement in actions is also partly determined through dopaminergic mechanisms associated with the assignment of positive valence, ensuring an alignment of incentive and activity [9–16].

By contrast, the case of aversive Pavlovian and instrumental conditioning is rather less well understood. Perhaps the most venerable puzzle concerns the instrumental case of active avoidance: how could it be that the desired absence of an aversive outcome can influence the choice and motivation of behaviour [17–22]? However, implementational considerations about the architecture of control make for extra problems—if, for instance, vigorous engagement in actions associated with active defence requires recruitment of mechanisms normally thought of as being associated with rewards rather than (potential) punishments [23, 24]. Further, there are alternative passive and active defensive strategies that impose seemingly opposite demands on these systems [25, 26].

In this review, we examine aversion through the medium of dopamine and some of its key targets. Dopamine is by no means the only, or perhaps even the most important, implementational facet of negative valence. For instance, as we will see, complex, species-specific, defensive systems provide an elaborate hard-wired mosaic of responsivity to a panoply of threatening cues [27–29]. Furthermore, cortically-based methods of reasoning that can incorporate and calculate with intricate prior expectations over such things as the degree to which environmental contingencies afford control, play a crucial role in modulating these defences [30–32]. Nevertheless, dopamine is well suited to the purpose of elucidating aversion because of the role it plays in the above enigmas via its influence over learned choice and vigour. Of dopamine’s targets, our principal focus here is the striatum, with particular attention to D_2_ receptors because of their seemingly special role in passive forms of behavioural inhibition [8, 33].

Almost all the elements of this account have been aired in previous analyses of appetitive and aversive neural reinforcement learning, with the role of dopamine also attracting quite some attention [34–40]. Our main aims are to weave these threads together, using the sophisticated view of appetitive conditioning as a foundation for our treatment of the aversive case, and to highlight issues that remain contentious or understudied. The issue of behavioural control will turn out to be key. We first outline a contemporary view of appetitive conditioning. We then use this to decompose and then recompose the issues concerning innate and learned defence.

## Prediction and control of rewards

Reinforcement learning (RL) addresses the following stark problem: learn to choose actions which maximize the sum of a scalar utility or reward signal over the future by interacting with an initially unknown environment. Such environments comprise states or locations, and transitions between these states that may be influenced by actions. What make this problem particularly challenging are both the trial-and-error nature of learning—the effect of actions must be discovered by trying them—and the possibility that actions affect not only immediate but also delayed rewards by changing which states are occupied in the future [41].

Two broad classes of RL algorithms address this computational problem: *model-based* and *model-free* methods [41, 42]. Briefly, model-based methods use experience to construct an internal model of the structure of the environment (i.e. its states and transitions) and the outcomes it affords. Prediction and planning based on the model can then be used to make appropriate choices. Assuming the possibility of constant re-estimation, the flexibility afforded by this class of methods to changes in contingency (i.e. to environmental structure) and motivational state (i.e. to outcome values) has led to the suggestion that it is suitable as a model of goal-directed action [43–46]. Model-based estimates can also encompass comparatively sophisticated ‘meta-statistics’ of the environment, such as the degree to which rewards and punishments are under the control of the agent [32].

By contrast, model-free methods do not construct an internal model, but rather learn simpler quantities in the service of the same goal. One such is the mean *value* of a state, which summarizes how good it is as judged by the cumulative rewards that are expected to accrue in the future when the subject starts from that state. This is, of course, the quantity that requires optimization. Crucially, the values of successive states satisfy a particular consistency relationship [47], so that states which tend to lead to states of high value will also tend to have high value, and vice-versa for states which tend to lead to low-value states. A broad class of model-free RL methods, known as *temporal difference* (TD) methods, use inconsistencies in the values of sampled successive states—a TD *prediction error* signal—to improve estimates of state values [48].

For selecting appropriate actions, a prominent model-free method is the *actor-critic* [41, 49]. This involves two linked processes. One is the *critic*, which uses the TD error to learn the model-free value of each state. However, future rewards typically depend on the actions chosen, or the behavioural *policy* followed. A policy is a state-response mapping, and is stored in the other component, the *actor*, which determines the relative probabilities of selecting actions. It turns out that the same TD prediction error that can improve the predictions of the critic may also be employed to improve the choices of the actor. There are also other model-free quantities that can be used for action selection. These include the *Q* value [50] of a state-action pair, which reports the expected long-run future reward for taking the particular initial action at the state.

Such model-free methods have the virtue of being able to learn to choose good actions without estimating a world model. However, summarizing experience by simple state values also means that these methods are relatively inflexible in the face of changes in environmental contingencies. Consequently, model-free RL methods have been suggested as a possible model of habitual actions [44, 45].

Both model-based and model-free methods must balance exploration and exploitation. The former is necessary to learn the possibilities associated with a novel domain; the latter then garners the rewards (or avoids the punishments) that the environment has been discovered to afford. This balance depends sensitively on many factors, including prior expectations about the opportunities and threats in the environment, how much control can be exerted over them, and how fast they change [51]. It also requires careful modelling of uncertainty—for instance, it is possible to quantify the value of exploration of unknown options as a function of the expected worth of the exploitation that they could potentially allow in the future [52, 53]. The excess of this over the expected value given current information is sometimes known as an *exploration bonus*, quantifying optimism in the face of uncertainty [54, 55].

Calculating such bonuses correctly, balancing exploration and exploitation optimally, and even just finding the optimal trajectory of actions in a rich state space, are radically computational intractable; heuristics therefore abound which are differently attuned to different classes of method [51]. Perhaps the most important heuristic is the existence of hard-wired systems that embody pre-specified policies. As we will detail below, these are of particular value in the face of mortal threat—animals will rarely have the luxury of being able to explore to find the best response. However, they are also useful in appetitive cases, obviating learning for actions that are sufficiently evolutionarily stable, such as in food-handling, mating and parenting.

Such hard-wired behaviours may be elicited in the absence of learning by certain stimuli, which are therefore designated *unconditioned stimuli* (USs). Presentation of a US typically inspires what is known as a *consummatory* response, attuned to the particularities of the US. It is through Pavlovian, or classical, conditioning that such innate responses can be attached not only to USs but also to formerly neutral predictors of such outcomes. These predictors are then called *conditioned stimuli* (CSs) since their significance is ‘conditioned’ by experience. Along with targeted preparation for particular outcomes, CS-elicited *conditioned responses* (CRs) include generic, so-called *preparatory*, actions: typically approach and engagement for appetitive cues, associated with predictions of rewarding outcomes; and inhibition, disengagement and withdrawal for aversive cues, associated with future threats or punishments. The predictions that underpin preparation can be either model-based or model-free [56]. We should note that the long-standing distinction between preparatory and consummatory behaviours [57–59] is not always clear cut; however, it has been usefully invoked—though not always in exactly the same terms—in various related theories of dopamine function [11, 60–66].

The fuller case of RL, in which actions come to be chosen because of their contingent effects rather than being automatically elicited by predictions, corresponds to instrumental conditioning. At least in experimental circumstances such as negative automaintenance [67], automatic, Pavlovian, responses can be placed in direct competition with instrumental choices. Perhaps surprisingly, Pavlovian responses often win [68, 69], leading to inefficient behaviour. A less malign interaction between Pavlovian and instrumental conditioning is called ’Pavlovian-instrumental transfer’ (PIT) [45, 70–73]. In this, the vigour of instrumental responding (typically for rewards) is influenced positively or negatively by the presence of Pavlovian CSs associated with appetitive or aversive predictions, respectively.

We start by considering the implications of Pavlovian and instrumental paradigms for the neural realization of control. We use a rather elaborated discussion of appetitive conditioning and rewards as a foundation, since this valence has received more attention and so is better understood. As a preview, we will see that dopamine in the ventral striatum has a special involvement in model-free learning (reporting the TD prediction error). However, dopamine likely also plays an important role in the expression and invigoration of both model-based and model-free behaviour.

## Predicting reward: Pavlovian conditioning

Model-free RL, with its TD prediction errors, has played a particularly central role in developing theories of how animals learn state values, the latter interpreted as the predictions of long run rewards that underpin Pavlovian responses [74–77]. There is by now substantial evidence that the phasic activity of midbrain dopamine neurons resembles this TD prediction error in the case of reward [40, 78, 79]. Neural systems in receipt of this dopamine signal are then prime candidates to represent state values. One particularly important such target is the cortical projection to the ventral striatum (or nucleus accumbens; NAc) [78, 80], the plasticity of whose synaptic efficacies may be modulated by dopamine [81–85]. Note that, by contrast, dorsomedial and dorsolateral striatum, which are also targeted by dopamine cells—though by cells in the substantia nigra (SNc) rather than in the ventral tegmental area (VTA)—have been associated respectively with model-based and model-free instrumental behaviour (see below).

Along with its involvement in plasticity, dopamine, particularly in the NAc, has long been implicated in the intensity of the expression of innate, motivated behaviours (i.e., just those behaviours elicited by Pavlovian predictions) in response to both unconditioned and conditioned stimuli [66, 86, 87]. This is a form of Pavlovian vigour [63, 64, 88–91]. Relevant CSs have been described as acquiring ‘incentive salience’ [65, 92] or ‘incentive motivation’ [93], possibly via the way that their onset leads to TD errors that reflect state predictions [94]. Perhaps also related to Pavlovian vigour is the observation that the influence of CSs on instrumental responding in PIT paradigms is sensitive to dopamine signalling too [95–97]. It has recently been shown that dopaminergic projections to ventral striatum corelease glutamate [98–100], though see [101], which may modulate these effects.

The influence of dopamine neurons over the expression of behaviour might extend to model-based as well as model-free predictions, based on other afferent projections to the dopamine system. Model-based values are thought to be stored in, and calculated by, other areas, such as the basolateral amygdala and orbitofrontal cortex [87, 102–109].

Three further details of the ventral striatum and dopamine release in this structure are important. Firstly, anatomically, the NAc is classically subdivided into ‘core’ (NAcC) and ‘shell’ (NAcS) subregions [110]. As well as being histochemically distinct, these regions differ in their patterns of connectivity. For example, while NAcC resembles dorsal striatum in projecting extensively to classic basal ganglia output structures, such as the ventral pallidum, NAcS is notable for its projections to subcortical structures outside the basal ganglia, such as lateral hypothalamus and periaqueductal gray (PAG), which are involved in the expression of unlearned behaviours [110–114].

Two related ideas are abroad about the separate roles of these structures. One is that NAcS and NAcC mediate the motivational impact of USs and CSs, respectively [87]. For instance, the projection of NAcS to the lateral hypothalamus is known to play a role in the expression of feeding behaviour [112], requiring intact dopamine signalling within NAcS [115]. Conversely, conditioned approach is impaired by lesions or dopamine depletion of NAcC, but not by lesions of NAcS [116, 117].

The other idea is that NAcS and NAcC are involved in *outcome-specific* and *general* PIT, respectively [118]. The difference concerns whether the Pavlovian prediction is of the same outcome as for the instrumental act (specific PIT), or instead exerts influence according to its valence (general PIT). It has been reported that lesions of NAcS abolished outcome-specific PIT but spared general PIT, while lesions of NAcC abolished general PIT but spared outcome-specific PIT [118].

These ideas are not quite compatible, since both sorts of PIT involve conditioned stimuli. Perhaps, instead, we should think of the NAcC as being more involved in preparatory behaviours, attuned only to the valence (positive or negative) of a predicted outcome but not its particularities, while the NAcS is more involved in consummatory behaviours, which additionally reflect knowledge of the particular expected outcome(s) [118–123]. This is less incompatible with the first idea than it might seem, since outcome-specific PIT presumably relies on representation of the US, even if the US itself is not physically present [56]. This latter interpretation aligns with the distinction between model-free and model-based RL predictions, which would then be associated with NAcC and NAcS, respectively [56].

The second relevant, if somewhat contentious (see below), feature is that, as appears to be the case in the striatum generally, the majority of the principal projection neurons in NAc—medium spiny neurons (MSNs)—may express either D_1_ or D_2_ receptors, but not both [124, 125]. Briefly, dopamine receptors are currently thought to come in five subtypes, each classified as belonging to one of two families based on their opposing effects on certain intracellular cascades: D_1_-like (D_1_ and D_5_ receptors), and D_2_-like (D_2_, D_3_, and D_4_ receptors). D_1_ and D_2_ receptors are of prime interest here since they are by far the most abundantly expressed dopamine receptors in the striatum and throughout the rest of the brain [126–128]. In the striatum, the majority of D_1_ and D_2_ receptors are thought to occupy states in which their affinities for dopamine are low and high respectively [129], with the consequence that these receptors are influenced differently by changes in phasic and tonic dopamine release [130]. Furthermore, D_1_ and D_2_ receptors appear to mediate opposite effects of dopamine on their targets: activation of D_1_ receptors tends to excite, and D_2_ to inhibit, neurons; this modulation of excitability can then also have consequences for activity-dependent plasticity [131, 132].

In the dorsal striatum, there is substantial evidence for an anatomical segregation between D_1_-expressing ‘Go’ (direct; striatomesencephalic) and D_2_-expressing ‘NoGo’ (indirect; striatopallidal) pathways [131, 133–136]. The effect of these pathways on occurrent and learned choice is consistent with the observations about the activating effect of dopamine [137], as we discuss in more detail below. Equivalent pathways are typically assumed to exist in NAc [138–140] although the segregation here seems more debatable [111, 141–143]. Indeed, D_1_-expressing MSNs within NAcC are reported to also project within the striatopallidal (‘indirect’) pathway [141, 144]; there is evidence for co-expression of D_1_ and D_2_ receptors, particularly in NAcS [124, 145, 146]; and there are suggestions that D_1_ and D_2_ receptors can interact to form heteromeric dopamine receptor complexes within the same cell [147, 148], though this appears to be still a matter of question [149]. In functional terms, though, at least in the case of appetitive conditioning, it seems there may be parallel Go and NoGo routes, given evidence that D_1_ receptors may be of particular importance in learning Pavlovian contingencies [150–154], while antagonists of either D_1_ or D_2_ receptors appear to disrupt the expression of such learning [155–159], including the expression of preparatory Pavlovian responses [34, 153]. Unfortunately, given the possible association of core and shell with model-free and model-based systems above, experimental evidence that clearly disentangles the roles of D_1_ and D_2_ receptors in these respective areas in appetitive conditioning appears to be lacking.

The third detail, which applies equally to ventral and dorsal striatum, concerns the link between the activity of dopaminergic cells and the release of dopamine into target areas. While there is little doubt that phasic release of striatal dopamine can be driven by activity in midbrain dopaminergic cells (e.g. [160]), a range of mechanisms local to the striatum is known to play a role in regulating dopamine release, including a host of other neurotransmitters such as glutamate, acetylcholine, and GABA (for recent reviews, see [161, 162]). Indeed, recent evidence suggests that striatal dopamine release can be stimulated axo-axonally by the synchronous activity of cholinergic interneurons, separate from changes in the activity of dopaminergic cells [163]. Furthermore, it has long been suggested that there is at least some independence between fast ‘phasic’ fluctuations in extracellular dopamine within the ventral striatum and a relatively constant ‘tonic’ dopamine level; the former are proposed to be spatially restricted signals driven by phasic bursting of dopamine cells, while the latter is thought to be comparatively spatially diffuse and controlled rather by the number of dopamine cells firing in a slower, ‘tonic’ mode of activity [164–166]. Evidence for co-release of other neurotransmitters alongside dopamine, such as glutamate and GABA, adds further complexity [98, 100, 167, 168].

## Controlling reward: instrumental conditioning

In the instrumental, model-free, actor-critic method, the critic is the Pavlovian predictor, associated with the ventral striatum. The actor, by contrast, has been tentatively assigned to the dorsal striatum [78, 80, 169] based on its involvement in instrumental learning and control [170, 171]. The dorsal striatum is also a target of dopamine neurons, albeit from the substantia nigra pars compacta (SNc) rather than the ventral tegmental area (VTA). At a slightly finer grain, habitual behaviour has been particularly associated with dorsolateral striatum [172–175], while goal-directed behaviour has been associated with dorsomedial striatum, as well as ventromedial prefrontal and orbitofrontal cortices (for recent reviews, see [176, 177]). Recent evidence implicates lateral prefrontal cortex and frontopolar cortex in the arbitration between these two different forms of behavioural control in humans [178], and pre- and infra-limbic cortex in rats [179].

As noted above, the classical view of dorsal striatum is that the projections of largely separate populations of D_1_-expressing (dMSNs) and D_2_-expressing (iMSNs) medium spiny neurons are organised respectively into a direct (striatonigral) pathway, which promotes behaviour, and an indirect (striatopallidal) pathway, which suppresses behaviour [133, 134]. This dichotomous expression of D_1_ and D_2_ receptors would then allow dopamine to modulate the balance between the two pathways by differentially regulating excitability and plasticity [131]. In particular, activation of D_1_ receptors in dMSNs increases their excitability and strengthens the direct pathway via long-term potentiation (LTP) of excitatory synapses. By contrast, activation of D_2_ receptors in iMSNs decreases their excitability and weakens the indirect pathway by promoting long-term depression (LTD) of excitatory synapses.

This effect is then the basis of an elegant model-free account of instrumental conditioning [137, 180–182]. The active selection or inhibition of an action is mediated by the balance between direct and indirect pathways. Phasic increases and decreases in dopamine concentration report whether an action results in an outcome that is better or worse than expected, either via direct delivery of reward, or a favourable change in state. An increase consequent on the outcome being better than expected strengthens the direct pathway, making it more likely that the action will be repeated in the future. By contrast, a decrease consequent on the action being worse than expected strengthens the indirect pathway, making a repeat less likely. Much evidence, including recent optogenetic results, appears to support this basic mechanism [181, 183], although it is important to note that recent results suggest a slightly more nuanced view of the simple dichotomy between direct and indirect pathways—for instance, they are reported to be coactive during action initiation [184], consistent with the idea that they form a centre-surround organisation for selecting actions [185–187].

While it is natural to associate a dopamine TD prediction error with model-free prediction and control, there are hints that this signal shows a sophistication which potentially reveals more model-based influences [56, 188–191]. One such influence is exploration: observations of phasic activity of dopamine neurons in response to novel input which is not rewarding in any obvious sense (e.g. a novel auditory stimulus [192]) have been considered as an optimism-based exploration bonus [193]. It is not clear whether such activations depend, as they normatively should, on factors such as reward/punishment controllability that are typically the preserve of model-based calculations. Further, there remains to be a clear analysis of the role dopamine plays in the dorsomedial striatum’s known influence over model-based RL [194, 195].

## Instrumental vigour

Along with Pavlovian vigour is the possibility of choosing the alacrity or force of an action based on the contingent effects of this choice. Dopamine has also been implicated in this [12], potentially associated with model-based as well as model-free actions [196].

One idea is that there is a coupling between instrumental vigour and relatively tonic levels of dopamine, in the case that the latter report the prevailing average reward rate [9, 197]. This quantity acts as an opportunity cost for sloth, allowing a need for speed to be balanced against the (e.g., energetic) costs of acting quickly. Experiments that directly test this idea have duly supported dopaminergic modulation of vigour in reward-based tasks [13, 14, 16]. Formally, the average rate of TD prediction errors is just the same as the average rate of rewards, suggesting that nothing more complicated would be necessary to implement this effect than averaging phasic fluctuations in dopamine, at least in the model-free case. It could then be that because phasic fluctuations reflect Pavlovian as well as instrumental TD prediction errors, vigour would also be influenced by Pavlovian predictions—something that is contrary to the original instrumental expectation [9] but which is apparent in cases such as PIT [95]. Tonic dopamine has, of course, been suggested to be under somewhat separate control from phasic dopamine [164–166].

The putative involvement of dopamine in both vigour and valence leads to the prediction of a particular sort of hard-wired misbehaviour, or Pavlovian-instrumental conflict, namely that it might be hard to learn to withhold actions in the face of stimuli that predict rewards if inhibition is successful. This is indeed true, for both animals [67] and humans [24].

## Defence

The main intent of this review is to understand how the elements of adaptive behaviour that we have just described apply in the aversive case. Coarsely, we need to (i) examine the complexities of consummatory versus preparatory, and active versus passive, defensive choices in the face of unconditioned aversive stimuli and their conditioned predictors; (ii) consider how instrumental avoidance actions can be learned to prevent threats from arising in the first place; and (iii) consider how the vigour of defensive actions is set appropriately.

The reason that we structured this review through the medium of dopamine is that it seems that many of the same dopaminergic mechanisms that we have just described for appetitive conditioning also operate in the aversive case, subject to a few added wrinkles. This makes for puzzles, both for aversion (how one could get vigorous defensive actions when only potential punishments are present and the reward rate is therefore at best negative) and for dopamine (why dopamine would apparently be released in just such purely aversive circumstances).

We argue that it is possible to generalize to these cases an expanded notion of *safety* (cf. [64]), which itself underpins the popular, two-factor solution to instrumental avoidance [17, 19–22, 198–201]. Amongst other things, this implies subtleties in the semantics of dopamine, and a need to pay attention to the distinctions between reinforcement versus reward, and better versus good. To anticipate, we suggest that evidence for positive phasic and tonic dopamine responses to aversive unconditioned and conditioned stimuli may be explained in terms of a prediction of possible future safety. Furthermore, we suggest that these dopamine responses, and the consequent stimulation of striatal D_2_ receptors in particular, play an important role in promoting, or at least licensing, active defensive behaviours.

## Aversive unconditioned stimuli

There is some complexity in the consummatory response to an appetitive unconditioned stimulus (US) depending on how it needs to be handled. However, the response elicited by an aversive US—notably fleeing, freezing, or fighting—appears to depend in a richer way on the nature of the perceived threat, and indeed the species of the animal threatened [27]. Different emphases on the nature of the threat, or ‘stressor’, and the defensive response, or ‘coping strategy’, have led to subtly different, yet complementary, analyses of defensive behaviour and its neural substrates, which include the amygdala, ventral hippocampus, medial prefrontal cortex (mPFC), ventromedial hypothalamus, and periaqueductal gray (PAG) [25, 26, 28, 202–208] (for a recent review, see [29]).

For our purposes, the most important distinction is between *active* defensive responses, such as fight, flight, or freeze, and *passive* ones, such as quiescence, immobility, or decreased responsiveness. These need to be engaged in different circumstances, subject particularly to whether or not the stressor is perceived as being escapable or controllable [25]. Thus, active responses are adaptive if the stressor is perceived as escapable, since these may cause the stressor to be entirely removed. Conversely, passive responses may be more adaptive in the face of inescapable stress, promoting conservation of resources over the longer term and potential recovery once the stressor is removed. In other words, active responses entail *engagement* with the environment, while passive responses entail a degree of *disengagement* from the environment [25]. Even freezing involves ‘attentive immobility’, which can be interpreted as a state of high ‘internal’ engagement in threat monitoring.

The potential link to dopamine here is the proposal, particularly advocated by Cabib and Puglisi-Allegra [209–211] and fleshed out below, that an increased tonic level of dopamine in NAc, and especially the resulting stimulation of dopamine D_2_ receptors in this area, promotes active defence, whereas a decreased tonic level of dopamine in NAc, and the resulting decrease in D_2_ stimulation, promotes passive defence. This suggestion has clear parallels in the appetitive case. As there, in addition to the canonical direct and indirect pathways, typically associated with dorsal striatum and the expression of instrumental behaviours via disinhibition of cortically-specified actions [133, 137, 185, 212], we should expect accumbens-related Pavlovian defence to involve disinhibition and release of innate behavioural systems organised at the subcortical level, such as in the hypothalamus and PAG [112, 204, 213, 214].

For dopamine release, studies using microdialysis to measure extracellular concentrations of dopamine have reported elevated levels in response to an aversive US in NAc [215, 216], as well as in PFC [217] and amygdala [218, 219]. Using the higher temporal resolution technique of fast-scan cyclic voltammetry (FSCV), it has been reported that an aversive tail pinch US immediately triggers elevated dopamine release in the NAcC which is time-locked to the duration of the stimulus, while in the NAcS dopamine release is predominantly inhibited during the stimulus and either recovers or exceeds baseline levels following US offset [220, 221].

The substrate for this release is less clear. As we noted, many dopamine neurons appear to be activated by unexpectedly appetitive events. Although most studies report that dopamine neurons are inhibited by an aversive US (e.g., an electric shock, tail pinch, or airpuff), there are long-standing reports suggesting that a relatively small proportion may instead be activated [40]. The dopaminergic nature of some such responses appears to have been confirmed more recently via optogenetics [222] and juxtacellular labelling [223]. It has also been suggested that a particular group of ventrally-located dopamine cells in the VTA that projects to mPFC [224, 225] is more uniformly excited by aversive USs [223, 226]. In the SNc, it has recently been reported that dopamine cells projecting to the dorsomedial striatum show immediate suppression of activity, followed by sustained elevation of activity, in response to a brief electrical shock. By contrast, dopamine cells projecting to dorsolateral striatum display an immediate increase in activity before promptly returning to baseline [227].

In relation to defensive behaviour, pharmacological interventions and lesion studies have long suggested that dopamine plays a role (reviews include [12, 34]). More recent evidence supporting a particular role for NAc D_2_ receptors in defence comes from a series of experiments exploiting the ability of local disruptions to glutamate signalling in NAcS to elicit motivated behaviours [228, 229]. Thus, Richard and Berridge [230] have shown that expression of certain active defensive behaviours in rats (escape attempts, defensive treading/burying), which can be elicited by local AMPA blockade caudally in medial NAcS, not only requires endogenous dopamine activity [115], but also intact signalling of both D_1_ and D_2_ receptors. By contrast, (appetitive) feeding behaviour, elicited by glutamate disruption more rostrally in the medial NAcS, only requires intact signalling of D_1_ receptors [230]. This result supports a role for D_1_ receptors in active defence—as well as particular subregions of NAcS (though see [231] for evidence that the behaviours elicited from these regions is sensitive to context)—but it also seems to indicate an asymmetry in the involvement of D_2_ receptors in modulating the expression of innate appetitive versus defensive behaviours.

Other studies also suggest a role for D_2_ stimulation in active defence, though do not necessarily trace this to NAcS. For example, the expression of certain defensive behaviours in cats (ear retraction, growling, hissing, and paw striking), elicitable by electrical stimulation in ventromedial hypothalamus, can also be respectively instigated or blocked by direct microinjection into that area of a D_2_ agonist or antagonist [232, 233]. Indeed, as mentioned previously, anatomical connections between NAcS and hypothalamus are known to play an important role in controlling motivated behaviours, with NAcS cast in the role of ‘sentinel’ allowing disinhibition of appropriate behavioural centres located in the hypothalamus [112, 214].

Such lines of evidence are consistent with promotion of active Pavlovian defences via enhanced dopamine release and increased NAc D_2_ stimulation. Evidence for the other side of the proposal—promotion of passive Pavlovian defences via a drop in dopamine release and reduced NAc D_2_ stimulation—is provided by experiments in which animals are exposed to chronic (i.e. inescapable) aversive stimuli, such as in animal models of depression [234]. Briefly, not only do animals in these settings show diminished expression of active defensive behaviours such as escape attempts over time [235–237], but it has also been observed that an initial increase in NAc tonic dopamine on first exposure to the stressor gradually gives way to reduced, below baseline, dopamine levels [238–241]. Since modifications of the animal’s behaviour over time in such cases are presumably driven by experience of the (unsuccessful) outcomes of its escape attempts, and so more naturally fit with an instrumental analysis, we postpone fuller discussion of these results until considering the issue of instrumental behaviour and controllability below. However, we note that these changes in patterns of defence and dopamine release over time potentially yield an interesting case of a model-based influence on dopamine and perhaps model-free behaviours.

Pain research provides a complementary view. Bolles and Fanselow [205] pointed out that efficacious (active) defence requires inhibition of pain-related behaviours oriented towards healing injuries. Thus, it was hypothesized that activation of a fear motivation system, which promotes defensive behaviours (i.e. fight, flight, or freeze), inhibits—for example, by release of endogenous analgesics—a pain motivation system, which promotes recuperative behaviours (i.e. resting and body-care responses). Similarly, activation of the pain system was hypothesized to inhibit the fear system since (active) defensive behaviours would interfere with recovery via (passive) recuperative behaviours. In this light, it is interesting to note the well-established link between NAc dopamine, and D_2_ stimulation in particular, and analgesia [242, 243]. Conversely, reductions in motivation in mouse models of chronic pain—consistent with energy-preserving, recuperative functions—have recently been shown to depend on adaptation of (D_2_-expressing) iMSNs in NAc [244], and that this adaptation includes an increase in excitability of iMSNs in medial NAcS [245]. In turn, these results are consistent with previous observations of reduced effortful behaviour caused by blockade of NAc D_2_ receptors [246, 247]. Both observations are consistent with reductions of actions involved in active defence being caused by the relative strengthening of a ventral indirect pathway.

While these various lines of evidence point to involvement of accumbens dopamine, and NAc D_2_ signalling in particular, in modulating defence, we note some important caveats. As mentioned earlier, the separation of direct and indirect pathways in the accumbens is subject to continuing debate, with evidence that D_1_-expressing MSNs in NAc also project within the canonical indirect pathway [141] and that a substantial proportion of NAc MSNs co-express D_1_ and D_2_ receptors [124]. Furthermore, while D_2_ receptors may be more attuned to changes in tonic dopamine levels by virtue of their higher affinity, such changes presumably affect occupancy at both D_1_ and D_2_ receptors dependent on their affinities [130]. In short, rather than completely separate D_1_ and D_2_ systems that can be independently switched on and off, the true situation is likely to be more complex. Furthermore, experiments involving dopamine receptor agonists and antagonists can be difficult to interpret, since they may involve certain side-effects—such as the well known extrapyramidal symptoms associated with D_2_ antagonists [248]—and placing the system into states not encountered during normal functioning.

From an RL perspective, the roles of dopamine and D_2_ receptors raise two salient issues. The first is how to make sense of the apparent asymmetry in the involvement of D_2_ receptors in defensive, as opposed to appetitive, behaviours. One possibility starts from the observation that traditional paradigms assessing the interaction of Pavlovian and instrumental conditioning suggest that the Pavlovian defence system is biased towards behavioural inhibition in the face of threat [249, 250]. This Pavlovian bias may potentially require relatively greater inhibition of the ventral indirect pathway in order to *disinhibit* active defensive responses when required. Of course, this mechanistic speculation merely poses the further question of why the Pavlovian defence system should be biased towards behavioural inhibition in the first place. One dubitable speculation is that this stems from asymmetries in the statistics of rewards and punishments in the environment [251]. However, more work is necessary on this point.

The second, and more fundamental, issue is how to interpret variation, particularly enhancement, of NAc dopamine release in response to an aversive US in the first place, given the apparent tie between dopamine, appetitive prediction errors, and reward rates. This is the extended version of the puzzle of active avoidance to which we referred at the beginning. To answer this, we first consider certain similarities and differences between the unexpected arrival of an appetitive or aversive US [252]. This requires us to be more (apparently pedantically) precise about the appetitive case than previously. Here, the unpredicted arrival of the appetitive US (e.g. food) represents an unexpected improvement in the animal’s situation. This improvement stems from the fact that the US predicts that an outcome of positive value is immediately attainable. Indeed, all USs can be thought of as predictors, where these predictions are not learned but rather hard-wired. Thus, as previously noted, an appetitive US will engage innate behaviours such as salivation and approach. In turn, these unconditioned responses can be interpreted as reflecting at least an implicit expectation that the predicted reward is attainable/controllable, or at least potentially so, subject to further exploration. Thus, salivation in response to the presence of a food US can be interpreted as reflecting a tacit belief that the food will be consumable (and both require and benefit from ingestion). As reviewed above, the phasic responses of dopamine cells in response to the unexpected presentation of an appetitive US, along with other observations, encourage a TD interpretation in terms of a response to an unexpected predictor of future reward.

Consider now the arrival of an unexpected aversive US (e.g. the sight of a predator). What this event signifies seems more complex. On the one hand, this surprising event presumably indicates that the present situation is *worse* than originally expected, since the animal is now in an undesirable state of danger: i.e., (a) the aversive US is an ‘unpredicted predictor of possible future punishment’. As such, we should expect a negative prediction error. Indeed, at least the net value of the prediction error had better be negative to avoid misassignment of positive values to dangerous states and the consequent development of masochistic tendencies (i.e., the active seeking out of such dangerous states). On the other hand, relative to this new state of danger, the possible prospect of future safety—a positive outcome—comes into play. That is, at the point that the animal would actually manage to eliminate the threat if it can do so, the change in state from danger to safety would lead to an appetitive prediction error—just as with the change in state associated with the unexpected observation of food. Thus, provided the animal has the expectation that it will ultimately be able to achieve safety, i.e., that the situation is controllable, observation of the aversive stimulus should predict this future appetitive outcome, and so (b) lead to an immediate appetitive prediction error. The challenge therefore seems to be that of reconciling (a) and (b), i.e., the role of the aversive US as unpredicted predictor of both danger and possible future safety. To avoid any confusion, note that we discuss learning processes associated with signalling safety below; here, we consider hard-wired assessments of the absence of danger.

One attractive reconciliation comes from appealing to the concept of *opponency* [59, 253, 254]. Here, an aversive process would ensure that the net TD error caused by the unexpected aversive US is negative and that dangerous states are correctly assigned negative value. At the same time, an appetitive process would motivate behaviour towards the comparatively benign state of safety. Indeed, it has previously been proposed that the net prediction error can be decomposed in exactly this way [255], with the phasic activity of dopamine neurons signalling the appetitive component of this signal, while the aversive component is signalled by other means (e.g. by phasic serotonergic activity [23, 249, 252, 256]), such that the net prediction error would actually be negative [252].

A further consideration is the value of exploration. In appetitive contexts, we noted that exploration can be motivated by bonuses associated with the future value of what might be presently discovered. A potential heuristic realization of this was through the phasic activity of dopamine neurons inspired by novel stimuli [192, 193]. Consider the extension of this logic to the unexpected arrival of an aversive US: the animal may have the pragmatic *a priori* belief that safety is controllable, but the unexpected (and therefore ‘novel’) arrival of an aversive US may nevertheless be attended by uncertainty about how this new situation should be controlled. The issue of how exploration may then be carried out in a benign manner is of course particularly salient here (for a recent view of the issue of safe exploration from the RL perspective, see, e.g. [257]). The idea that a novel stressor elicits exploration in the ‘search for effective active coping’ has also been suggested by Cabib and Puglisi-Allegra [211]. In their scheme, a novel stressor leads to release of noradrenaline in PFC and dopamine in NAc; both of these are hypothesized to contribute to an active coping response by encouraging exploration (noradrenaline in PFC) and active removal of the stressor (stimulation of D_2_ receptors in NAc). Of particular note is that insufficient exploration can lead to persistent miscalibration [258]. That is, if the subject fails to explore, for instance because it believes the aversive stimulus to be insufficiently controllable, then it would never discover that it actually might be removed. Such a belief could result from a computational-level calculation about generalization from prior experience (as in learned helplessness; [31, 32]). At a different level of explanation, insufficient stimulation of D_2_ receptors, leading to a lack of inhibition of passive defensive mechanisms, could readily have the same consequence.

Relevant to the issue of exploration and dopamine’s possible involvement is the topic of anxiety. Fear and anxiety can be differentiated both by the behaviours they characteristically involve and their sensitivity to pharmacological challenge [259, 260]. Experimental assays of anxiety typically involve pitting the motivation to approach/explore novel situations against the motivation to avoid potential hazards [261]. According to one influential theory, it is exactly the function of anxiety in such cases of approach-avoidance conflict to move the animal towards potential danger, the better to assess risk [26, 259]. Not only is this thought to involve suppression of incompatible defensive responses, but also stimulation of approach; the associated ‘behavioural approach system’ is associated with NAc and its modulation by dopamine [259]. It would be interesting to consider a recent Bayesian decision-theoretic view of anxiety [262] that focuses on the opposite aspect, namely behavioural inhibition when there is no information to be gathered, and consider potential anti-correlations with dopaminergic modulation of the NAc.

In addition to evidence that some dopamine cells show phasic excitation in response to an aversive US, we also noted evidence from microdialysis studies for enhanced dopamine release in response to an aversive US over longer periods of time. What is the aversive parallel of the suggestion in the appetitive case that tonic dopamine levels, particularly in NAc, reflect an average reward rate which realizes the opportunity cost for acting slowly [9]? In aversive situations, the average reward rate is never strictly positive but, at least intuitively, time spent not actively engaged in a course of appropriate defensive action could be very costly indeed. For example, if an animal has just detected the presence of a predator, time spent not engaged in a course of defensive action could cost the potential safety that has thereby been missed.

Such considerations indicate the incompleteness of this previous account of tonic dopamine levels. In particular, dovetailing with our suggestions regarding phasic dopamine above, the suggested mapping of tonic dopamine to the average rate of reward needs to be broadened to include the potentially-achievable rate of safety [252] which, assuming a prior expectation of controllability, will be positive. This provides a possible explanation for why increased tonic dopamine concentrations have been observed in microdialysis studies in response to an aversive US. However, if the aversive US is inescapable or uncontrollable, then the potentially-achievable rate of safety reduces to nothing. Thus, the tonic release of dopamine would also be expected to decrease. This is consistent with evidence already mentioned that the initial increase in tonic NAc dopamine level dissipates over time, giving way to an eventual fall below baseline levels [238–241].

## Pavlovian conditioned defence

In relation to conditioning in aversive settings, similar complexities arise due to the fact that learning is likely to result in both aversive (i.e. danger-predicting) and appetitive (i.e. safety-predicting) conditioned stimuli, and may promote passive or active defensive strategies. Again, we use dopamine as a medium through which to view these complexities, with its preferential attachment to single sides of these dichotomies.

### Fear conditioning

Conditioning in the aversive case, where animals are exposed to cues predictive of aversive outcomes, is generally known as *fear conditioning* due to the constellation of physiological and behavioural responses that the aversive CS comes to evoke. As in the appetitive case, conditioned and unconditioned responses need not be the same. Take, for instance, the case of conditioning a rat to a footshock US [263]. Here, the predominant response of the rat on exposure to the environment where it has received footshocks in the past, i.e. the CR, is to freeze. By contrast, the immediate response elicited by the shock itself, i.e. the UR, is a vigorous burst of activity. Furthermore, there can be model-based, outcome-specific, predictions allowing tailored responses (e.g., [264]) as well as model-free, outcome-general, predictions leading to generic preparatory responses such as behavioural inhibition.

The intricacies of how CR and UR relate to each other, which are arguably greater in the case of fear conditioning where these may be in conflict, may explain some of the difficulties in explicating dopamine’s role in fear conditioning. A role for dopamine in fear conditioning seems to be generally accepted, though there is less consensus on the exact nature of this role (reviews include [265–267]).

Electrophysiological studies report that a substantial fraction (35–65 %) of putative dopamine neurons are activated by an aversive CS which is interleaved with an appetitive CS, a fraction that even exceeds the frequency (<15 %) of activations in response to an aversive US [191]. However, it has been suggested that many, though not all, of these activations may reflect ‘false aversive responses’, arising principally from generalization from appetitive to aversive CSs of the same sensory modality [191]. Additionally, an aversive CS may allow the animal to reduce the impact of an aversive US or avoid it entirely, and so in effect act as an instrumental ‘safety signal’, predicting a relatively benign outcome given a suitable defensive strategy. For example, a CS which predicts an aversive airpuff may facilitate a well-timed blink, thereby reducing the airpuff’s aversiveness [268]. This fits with the idea, mentioned above, that dopaminergic responses may be instigated by predicted safety, or a relative improvement in expected state of affairs.

Regardless of the interpretation of such activations of dopamine cells by aversive CSs, this activity appears to play a role in fear conditioning. For example, Zweifel et al. [269] have recently shown that disruption of phasic bursting by dopamine neurons via inactivation of their NMDA receptors impairs fear conditioning in mice. These mice apparently develop a ‘generalized anxiety-like phenotype’, which the authors ascribe to the animals’ failure to learn the correct contingencies.

Similar to observations in microdialysis studies of an increase in NAcS dopamine following an aversive US, enhanced NAcS dopamine release is also observed following presentation of an aversive CS [216]. Such enhanced release in NAcS to the onset of an aversive CS is corroborated by a recent FSCV study [270], though the opposite effect—decreased release—was observed in NAcC. Another recent FSCV study suggests that whether an increase or decrease in NAcC dopamine release is observed following an aversive CS depends critically on the animal’s ability to avoid the predicted US [271]. Thus, Oleson et al. [271] found that, when trained in a fear conditioning paradigm—where the aversive US (a shock) was necessarily inescapable—presentation of the CS led to a decrease in NAcC dopamine. By contrast, in a conditioned avoidance paradigm—where the animal could potentially avoid the shock—both decreases *and* increases in NAcC dopamine were observed: an *increase* on trials in which animals successfully avoided shock, but a *decrease* on trials in which animals failed to avoid shock.

Dopamine receptor subtypes appear to play distinct roles. There is some consensus that D_1_ receptor agonists and antagonists respectively promote or impede learning and expression in fear conditioning paradigms, while the effect of D_2_ manipulations is less clear [265, 267]. One study found that fear-potentiated startle could be restored in dopamine-deficient mice by administration of L-Dopa immediately following fear conditioning, but required intact signalling of D_1_ receptors but not of D_2_ receptors (although other members of the D_2_-like family of receptors were reportedly required; [272]). Consistent with this finding, it has been reported recently that striatal-specific D_1_ receptor knock-out mice, but not striatal-specific D_2_ receptor knock-out mice, exhibit strongly impaired contextual fear conditioning [273]. Combined with evidence from previous fear conditioning studies [267, 274–277], as well as extensive evidence from the conditioned avoidance literature (see below), it appears that D_2_ receptor manipulations affect only the *expression* of conditioned fear, rather than the learning of the association between aversive CS and US. This is consistent with experimental results in appetitive Pavlovian conditioning reviewed above, which suggest that D_1_ receptors are particularly important in learning the CS-US contingency, while both D_1_ and D_2_ receptors are involved in modulating expression of this learning. Further, it has been reported recently that disruption of dopamine signalling in NAcC, but not NAcS, attenuated the ability of an aversive CS to block secondary conditioning of an additional CS, suggesting differential involvement of these areas [278].

### Safety conditioning

The situation in which a CS predicts the *absence* of a US is usually known as ‘conditioned inhibition’ [279, 280]. In the particular case where the predicted absence is of an aversive US, the CS is called a *safety signal* [281, 282]. In considering aversive USs, we previously discussed hard-wired signals for safety—i.e., the absence of danger or threat. By contrast, here we consider previously neutral stimuli whose semantic association with safety is learned.

Such safety signals are capable of inhibiting fear and stress responses, and are known to have rewarding properties. For example, safety signals have been shown to act as conditioned reinforcers of instrumental responses [283]. This is consistent with the proposal of Konorski [59] and subsequent authors [199, 284, 285] that aversive and appetitive motivation systems reciprocally inhibit each other. The idea is that inhibition of the aversive system by a safety signal leads to disinhibition of the appetitive system, and so a safety signal is functionally equivalent to a CS that directly excites the appetitive system.

Neuroscientific study of safety signals is, however, at a relatively early stage (for reviews, see [281, 282]). Studies have identified neural correlates of learned safety in the amygdala [286–288] and striatum [286, 289]. Involvement of dopamine within NAcS in mediating the ability of the safety signal to inhibit fear, and consequently its ability to act as a conditioned reinforcer, is suggested by a recent study [290]. In particular, it was found that both infusion of d-amphetamine, an indirect dopamine agonist, and blockade of D_1_/D_2_ receptors in NAcS—but not in NAcC—disrupted the fear-inhibiting properties of a safety signal. While this finding implicates a role of NAcS in mediating the impact of the safety signal, why these manipulations had similar, as opposed to contrasting, effects is not clear.

## Instrumental defence: learning to avoid

The final form of learning we consider in detail is instrumental avoidance. This is a rich paradigm that involves many of the behaviours and learning processes that we have discussed so far: innate defence mechanisms, fear conditioning, safety conditioning, and instrumental learning (cf. [291]). Furthermore, a role of dopamine in active avoidance, and D_2_ receptors in particular, has long been suggested by the fact that dopamine antagonists interfere with avoidance learning [34, 292]. Indeed, such interference led to this paradigm being used to screen dopamine antagonists for antipsychotic activity [10, 12, 248]. Finally, the two-factor theory of active avoidance [17, 200, 201] that we discuss below was actually the genesis of the explanation we have been giving for the ready engagement of dopamine in the case of aversion.

### The problem of avoidance and two-factor theory

A typical avoidance learning experiment involves placing an animal (e.g., a rat) in an environment in which a warning signal (e.g., a tone) predicts future experience of an aversive US (e.g., a shock) unless the animal performs a timely instrumental avoidance response (e.g., shuttling to a different location, or pressing a lever). That animals successfully learn to avoid under such conditions posed a problem that concerned early learning theorists [18]: how can the nonoccurrence of an aversive event—a ubiquitous condition—act as a behavioural reinforcer?

A solution to this ‘problem of avoidance’ has long been suggested in the form of a two-factor theory [17, 59, 200, 293–296]. The name ‘two-factor’ refers to the hypothesis that two behavioural factors or processes—Pavlovian and instrumental—are involved in the acquisition of conditioned avoidance. Firstly, the warning signal comes to elicit a state of fear through its predictive relationship with the aversive US. Thus, the first factor of the theory refers to the Pavlovian process of fear conditioning. This Pavlovian process then allows the second factor to come into play: if the animal now produces an action leading to the cessation of the warning stimulus, the animal enters a state of relief, or reduced fear, capable of reinforcing the avoidance response. Thus, the second factor refers to an instrumental process by which the avoidance response is reinforced through fear reduction or relief. Such an account can also include stimuli dependent on the avoidance response and which are anticorrelated with the aversive US, thereby becoming predictive of safety [201]. As discussed, these safety signals (SS) themselves are thought to be capable of inhibiting conditioned fear [280], thereby both preventing Pavlovian fear responses (e.g., freezing) which may interfere with the instrumental avoidance response and reinforcing safety-seeking behaviours in fearful states or environments [294, 296], consistent with theories of opponent motivational processes [59, 285].

### Avoidance, innate defence, and controllability

As mentioned above, the importance of innate defensive behaviours in the avoidance context has long been noted. Bolles [27], highlighting the importance of such ‘species-specific defense reactions’, argued that if an avoidance behaviour is rapidly acquired, this is because the required avoidance response coincides with the expression of an innate defensive response by the animal, rather than reflecting a learning process; how difficult the animal finds the avoidance task will depend on the extent to which the avoidance response is compatible with its innate defensive repertoire. In turn, which innate behaviour the animal selects will be sensitive to relevant features of the avoidance situation, such as whether there is a visible escape route or not, reminiscent of Tolman’s [297] notion of behavioural support stimuli [206, 298].

Just as in the appetitive case, conflict between such Pavlovian behaviours and instrumental contingencies can lead to apparently maladaptive behaviour, albeit in rather unnatural experimental settings. Thus, Seymour et al. [299] highlight experiments in which self-punitive behaviour arises when an animal is (instrumentally) punished for emitting Pavlovian responses in response to that punishment. In one such unfortunate case, squirrel monkeys were apparently unable to decrease the frequency of painful shocks delivered to them by suppressing their shock-induced tendency to pull at a restraining leash attached to their collar; pulling on the restraining leash was exactly the action that hastened arrival of the next shock [300].

Similarly, just as it has been suggested that the animal’s appraisal of whether a threat is escapable or not is crucial in determining its defensive strategy in general (e.g., [25]), it was famously shown that the controllability of an aversive US is crucial in determining subsequent avoidance learning performance [301, 302]. In particular, dogs exposed to inescapable shocks in a first environment showed deficits in initiating avoidance or escape responses in a second environment, even though the aversive US was now escapable. This, of course, led to the concept of ‘learned helplessness’ [237]. Huys and Dayan [32] presented a model-based account of learned helplessness, arguing that the generalization between environments affected the value of exploration, thereby leading to persistent miscalibration.

The issue of model-free versus model-based influences has received rather less attention in aversive than appetitive contexts. However, sensitivity to revaluation of aversive USs in the context of instrumental avoidance has been demonstrated in rats [303, 304] and humans [305, 306], indicating model-based influences under at least some avoidance conditions. Fernando et al. [303] have recently reported that revaluation of a shock US induced by pairing shock with systemic analgesics (morphine or D-amphetamine), leading rats subsequently to decrease their rate of avoidance responding, could also be achieved by pairing the shock with more selective infusions of a mu-opioid agonist into either NAcS or PAG. Involvement of NAcS and related structures in revaluation in this instance is consistent with the idea that the shell is involved in model-based prediction [56].

### Dopamine, D_2_ receptors, and active avoidance

Two-factor theories of avoidance fit well with the idea that the striatum, in interaction with dopamine, implements an actor-critic algorithm [21, 22, 78, 80, 169, 307]. Thus, an initial period of learning by the critic (in the ventral striatum) of negative state values (i.e., fear conditioning) allows subsequent instrumental training of an avoidance response by the actor (in the dorsal striatum), since actions leading to the unexpected non-delivery of the aversive US are met with a positive prediction error (‘better than expected’), as signalled by dopamine neurons in the midbrain.

It was the abilities of certain antipsychotic drugs and neurotoxic lesions to produce active avoidance learning deficits [248, 292] that suggested a critical role for dopamine in the acquisition of conditioned avoidance. Furthermore, localised neurotoxic lesions suggested that dopamine projections to both dorsal and ventral striatum were required for acquisition of active avoidance [308, 309], corroborated by more recent work on selective restoration of dopamine signalling in dopamine-deficient mice [310]. This is consistent with complementary roles of actor (ventral striatum) and critic (dorsal striatum) in adapting behaviour.

Dopamine’s action on D_2_ receptors appears of particular importance for this. Evidence from active avoidance studies suggests that while blocking D_2_ receptors leaves fear conditioning intact, instrumental learning of the avoidance response requires intact D_2_ signalling [292, 311, 312]. From the perspective of the actor-critic, one might conclude that blockade of D_2_ receptors therefore does not interfere with the learning of negative state values by the critic but does interfere with the learning of the actor [22]. The finding that D_2_ receptor blockade leaves conditioning to aversive stimuli intact in the active avoidance setting is consistent with evidence from fear conditioning studies (see above). Furthermore, that D_2_ blockade also disrupts instrumental learning is consistent with dopamine’s modulation of direct and indirect pathways in the dorsal striatum, as in Frank’s [137] model, since this would be expected to lead to a relative strengthening of the indirect, ‘NoGo’ pathway and impede acquisition of the appropriate ‘Go’ response (albeit leaving this model without a means of implementing the preserved fear conditioning). However, this would raise the question of why D_2_ receptors within dorsal striatum should be implicated more strongly in learning than are those in ventral striatum. A pertinent observation might be a distinction between the longevity of the effects of tying optogenetic stimulation of (D_1_-expressing) dMSNs and (D_2_-expressing) iMSNs in the dorsomedial striatum [183] of mice. These authors triggered activation of one or other pathway when the mouse made contact with one of two touch sensors. dMSN stimulation increased preference for its associated lever, whereas iMSN stimulation decreased it. However, whereas the positive preference persisted in extinction throughout a test period, the negative preference rapidly disappeared. Furthermore, it was noted that stimulation of iMSNs elicited brief, immediate freezing followed by an ‘escape response’, though these behavioural changes were not thought sufficient to explain the bias away from the laser-paired trigger.

Nevertheless, while many findings accord well with an actor-critic account of avoidance learning, there are at least two omissions in such accounts that require correction. Firstly, similar to Bolles’ complaints about two-factor theory, actor-critic accounts have largely ignored the role for innate (i.e., Pavlovian) defence mechanisms. Secondly, the key factor of controllability has not been fully integrated with actor-critic models.

Indeed, disruption of innate defensive behaviour by D_2_ blockade occurs as well as disruption of instrumental learning of the active avoidance response. There are suggestions that suppression of conditioned avoidance may rely more on disruption of D_2_ signalling within ventral, rather than dorsal, striatum [248], consistent with interference with Pavlovian (‘critic’) rather than instrumental (‘actor’) processes. For example, post-training injection of a D_2_ antagonist into NAcS, but not into dorsolateral striatum, leads to a relatively immediate suppression of a conditioned avoidance response [313]. As we saw above, NAcS, under dopaminergic modulation, is implicated in controlling expression of innate defensive behaviours, and D_2_ activation appears to promote active defensive strategies. Similarly, there is evidence that D_2_ blockade leads to enhanced freezing responses—arguably, a more passive form of defence—following footshock, interfering with rats’ ability to emit avoidance responses [34, 274], though there remains some doubt about whether fear-induced freezing is an important factor in the disruption of conditioned avoidance [248]. In their review of the role of dopamine in avoidance learning, and defence more generally, Blackburn and colleagues [34] suggest that D_2_ blockade does not disrupt defensive behaviour globally but rather ‘changes the probability that a given class of defensive response will be selected’ ([34], p. 267), in particular increasing the probability of freezing.

In relation to controllability, we have already referred to evidence that exposure to chronic, inescapable stress abolishes stress-induced increases in the concentration of accumbens dopamine [238–241]. Such evidence has led Cabib and Puglisi-Allegra [209–211] to suggest that whether an increase or decrease in accumbens dopamine levels is observed in response to stress depends on whether the stressor is appraised as controllable (increase) or not (decrease). This dissipation of the dopamine response does not appear to be explained by dopamine depletion, since subsequent release from the chronic stressor leads to a large, rapid increase in dopamine concentration [239]. Similarly, Cabib and Puglisi-Allegra [209], using a yoked paradigm in which one of a pair of animals (the ‘master’) has some control over the amount of shock experienced by means of an escape response while the other (‘yoked’) animal does not, found evidence consistent with elevated and inhibited NAc dopamine in master and yoked animals, respectively, after an hour of shock exposure.

More recently, Tye et al. [314] used optogenetics to assess the effects of exciting or inhibiting identified VTA dopamine cells in certain rodent models of depression involving inescapable stressors (tail suspension, forced swim, and chronic mild stress paradigms). While optogenetic inhibition of these dopamine cells could induce behaviour that has been related to depression, such as reduced escape attempts, optogenetic activation of the same cells was found to rescue depression-like phenotypes (e.g., promoting escape-related behaviours) induced by chronic stress. Furthermore, it was observed that chronic stress led to a reduction in measures of phasic VTA activity. This latter observation contrasts with studies using repeated social defeat stress, where phasic VTA activity has typically been observed to increase in ‘susceptible’ animals [315–317]. Apparently contradictory findings regarding stress-induced changes in VTA dopamine activity, and indeed the effects of manipulating this via optogenetic stimulation, might stem from the subtleties of the different paradigms used, but may also reflect heterogeneity in the properties of different VTA dopamine cells, such as between those projecting to mPFC versus NAc (for a recent discussion of these issues, see [318]).

While there is evidence from microdialysis studies that support a link between controllability, defensive strategy, and tonic NAc dopamine, it should be noted that not all such evidence points in this direction. For example, Bland et al. [319] measured both dopamine and serotonin release in NAcS of rats in the yoked pairs paradigm referred to previously. While they did report a trend for increased dopamine release relative to no-shock controls, this increase was neither significant nor differed between master and yoked animals. By contrast, serotonin levels were found to be significantly increased in yoked animals during and after stress exposure, relative to master and no-shock control animals [319]. Experiments using the same paradigm but taking measurements from mPFC found elevated levels of both dopamine and serotonin in yoked animals compared to master and no-shock controls [320].

These latter studies and others [321–323] highlight that consideration of other neuromodulators, notably serotonin, is crucial for a fuller understanding of defensive behaviour. A role of serotonin has long been suggested both in the particular case of active avoidance [312, 324] and in defence more generally [249, 250]. As mentioned, one suggestion is that the putative opponency between appetitive and aversive motivation systems [59, 254, 285] is at least partly implemented in opponency between dopamine and serotonin, respectively [23, 249–251, 256]. A specific computational model of this idea was suggested by Daw et al. [255], and Dayan [252] has more recently considered such opponency in the particular case of active avoidance. However, a modulatory role of controllability in the active avoidance setting has not yet been fully integrated into RL models.

## Conclusions

Here, we have discussed unconditioned/conditioned, Pavlovian/instrumental, and passive/active issues associated with aversion. We used dopamine, and particularly its projection into the striatum and the D_2_ system, as a form of canary, since the way that dopamine underpins model-free learning, and model-free and model-based vigour, turns out to be highly revealing for the organization of aversive processing. Our essential explanatory strategy rested on three concepts: safety, opponency, and controllability.

When under threat, safety is a desirable state. We suggested that the prospect of possible future safety underlies positive dopamine responses—both tonic and phasic—in response to aversive stimuli. Indeed, the interpretation of these responses is very similar to the more obviously appetitive case involving rewards, since safety is an appetitive outcome. Thus, phasic activation of dopamine cells in response to an aversive stimulus can be interpreted in TD terms as an ‘unpredicted predictor of future safety’. Similarly, increased levels of tonic dopamine in conditions of stress, particularly in NAc, can be interpreted as signalling a potentially-achievable rate of safety.

Of course, what makes safety a more subtle concept is that it is relative; it is defined in opposition to danger. Dangerous states are not, in general, good states, which is why, in opposition to an appetitive process directed at safety in such states, there should be an aversive system which signals the disutility of occupying dangerous states. Therefore, positive dopamine responses which putatively signal the appetitive component of a TD prediction error in such states can only be part of the story—an *opponent* signal is required, marking the value of the path that will (hopefully) not be taken, and providing a new baseline against which to measure outcomes. This results in a form of counterfactual learning signal, a quantity that has also been investigated in purely appetitive contexts, and may have special relevance to the dorsal, rather than the ventral, striatum [325–328].

Unfortunately, while the notion of opponent appetitive and aversive processes is long-standing [59, 253, 254, 285], we still know relatively little about their neural realization. As mentioned, one idea is that this opponency maps to dopamine (appetitive) and serotonin (aversive) signalling [23, 249–251, 256], and specific computational models of this idea have been advanced [252, 255]. Recent attention to electrophysiological recordings from identified serotonergic cells in conditions of reward and punishment is particularly welcome in this regard, albeit offering no comfort to these theoretical ideas [329], and we look forward to further work which leverages advances in neuroscientific techniques to clarify the neural substrate of opponency.

Whether safety is appraised as achievable or not appears to be crucial, hence our appeal to the concept of controllability. We reviewed evidence that tonic levels of dopamine are modulated downwards over time with chronic exposure to aversive stimuli. Further, we reviewed evidence that dopamine, and NAc D_2_-receptor stimulation in particular, modulates active versus passive defensive strategies (or perhaps better, defensive versus recuperative behaviours). Modulation of dopamine in this way raises pressing questions about controllability at both more and less abstract levels. Indeed, even formalizing an adequate concept of behavioural control in the first instance is nontrivial [32].

The concept of controllability brings model-based and model-free considerations back into focus since, at least intuitively, this concept seems to imply explicit knowledge of action-outcome statistics in the current environment. In relation to dopamine, this is consistent with evidence that a model-based system could potentially influence model-free learning and performance via the dopaminergic TD signal. However, implementation of heuristics aimed at optimizing the exploration-exploitation trade-off, such as possibly instantiated in a dopaminergic exploration bonus, may provide a model-free proxy for controllability. Thus, further work is required to disentangle the relative contributions of model-based and model-free systems in modulating dopamine signals which, in turn, modulate defensive strategy.

We have focused on dopamine in the accumbens at the expense of other areas—notably the amygdala and mPFC—which are of clear relevance to the themes discussed. For example, intact dopamine signalling in the amygdala, as well as in the striatum, appears to be necessary for acquisition of active avoidance behaviour [310], with the central nucleus particularly implicated in mediating conditioned freezing responses that may interfere with active responding [330]. Indeed, there is evidence that D_2_ receptors are particularly prevalent in the central amygdala [265, 331], and a recent review [332] suggests that a key role of D_2_ receptors in the central nucleus is to modulate reflex-like defensive behaviours organised in the brain stem. This clearly relates to the proposed importance of D_2_ in modulating Pavlovian defence discussed here. Similarly, it is known that stress-induced increases in accumbens dopamine release is constrained by activation of D_1_ receptors in mPFC, with both mPFC dopamine depletion or blockade of D_1_ receptors leading to enhanced stress-induced accumbens release of dopamine (see [211], and references therein). Furthermore, mPFC is thought to be a key player in the appraisal of whether a stressor is under the animal’s control [323].

Throughout the review, we have highlighted various issues that merit experimental and theoretical investigation. Experimentally, the most pressing issue is perhaps heterogeneity in the dopamine system—arriving at a clearer view of the potentially separate roles and activation of different groups of dopamine neurons, and reconciling activation and release. Technical advances have allowed increasingly sophisticated attacks on this issue, though a consensus regarding the degree of heterogeneity, both in terms of activity [333, 334] and connectivity [227, 335, 336], has yet to emerge. To the extent that dopamine neurons with different affective receptive fields project to different targets, there is no need for a shared semantics for their activation [23]. However, if reward and punishment-activated dopamine neurons are interdigitated in the way suggested by some experiments [333], then there is a need for a functional analysis as to how downstream systems might be able to interpret the apparently confusing patterns of dopamine release. One speculation in the former case, e.g. if dopamine cells in the ventral versus dorsal VTA showing different responses to aversive stimuli [223] also differentially project to more ventral versus dorsal regions of striatum, respectively, is that this reflects competing objectives to (a) shape (instrumental) policy *retrospectively*, by assigning (dis)credit to actions that may have led to aversive outcomes, and (b) to promote suitable (Pavlovian) behaviour *prospectively* in light of possible future safety.

Similarly, it would be important to understand the true degree of separation between putative direct and indirect pathways in the core and shell of the accumbens. Heterogeneity in the serotonin system, and its interactions with dopamine in the case of aversion, would also merit investigation. A recent revealing analysis of active avoidance in the zebrafish, showing the critical involvement of a pathway linking the lateral habenula to the median raphe [324] is of importance, particularly since most of the recent studies of optogenetically tagged or manipulated 5-HT neurons have focused instead on the dorsal raphe [329, 337–339]. Integrating the whole array of data on patience, satiety, motor action, behavioural inhibition and aversion associated with 5-HT is a major task.

From a more behavioural viewpoint, it would be interesting to get a clearer view of the scope of model-based aversive conditioning. For instance, take the experiment showing that D_2_ blockade does not arrest learning aversive predictions even though it does avoidance responses [311]: it is not clear why model-based predictions would not be capable of generating appropriate avoidance behaviour as soon as the D_2_ antagonist is washed out—rather leaving it to be acquired slowly, as if it was purely model-free.

Another important experimental avenue is to try and integrate the processing of costs (and indeed, for humans, outcomes such as financial losses) with that of actual punishment. Costs, which could be either physical or mental [340–342], also exert a negative force on behaviour, and indeed also have a slightly complicated relation to dopamine activation and release [12, 16, 343, 344].

From a more theoretical viewpoint, perhaps the most urgent question concerns pinning down the different facets of controllability, the way that these determine operations such as exploration, and relative model-based and model-free influences. Entropy and reachability of outcomes were considered by [32], but other definitions are possible. Work on learned helplessness suggests a key role for the mPFC in suppressing otherwise exuberant 5-HT activity in animals who have the benefit of behavioural control—but what exactly mPFC is reporting is unclear.

A further direction is to construct a more comprehensive theory of aversive vigour [252] looked at this in a rather specific set of experimental circumstances. The direct predictions arising even from this have not been thoroughly tested; but a more general theory, also tied to controllability, would be desirable.

Finally, we have noted various structural asymmetries between appetitive and aversive systems, ascribing many of them to asymmetric priors about the structure of rewards and punishments in environments [23]. It would be important to examine these claims in more detail, and indeed look at the effect of changing the statistics of environments to determine the extent of lability.

In conclusion, we have attempted to use our evolving and rich view of the nature and source of learned, appetitive behaviour to examine the case of aversion and defence. Along with substantial commonalities between the two, we have discussed some critical differences—notably in the way that aversive behaviour appears to piggy-back on appetitive processing, leading to various intricate complexities that are incompletely understood. Dopamine plays a number of critical and apparently confounded roles; we therefore used it to lay as bare as possible the extent and limits of our current understanding.
